# Identification of key genes and metabolites in acute ischemic stroke via integrated transcriptomic and metabolomic analysis

**DOI:** 10.3389/fneur.2026.1767562

**Published:** 2026-04-15

**Authors:** Jiayan Zhang, Xin Zhou, Dian He

**Affiliations:** Department of Neurology, Affiliated Hospital of Guizhou Medical University, Guiyang, Guizhou, China

**Keywords:** acute ischaemic stroke, biomarkers, enrichment analysis, key genes, metabolite

## Abstract

**Background:**

Acute ischaemic stroke (AIS) remains a leading cause of death and disability. This exploratory study integrated transcriptomic and metabolomic analyses to identify candidate hub genes and metabolites associated with AIS and to provide preliminary insights into its molecular mechanisms and potential therapeutic targets.

**Methods:**

Metabolomic sequencing data were analyzed using orthogonal partial least squares discriminant analysis (OPLS-DA), together with multivariate and univariate analyses, to identify differentially expressed metabolites (DEMs). Differential gene expression analysis was performed using the GSE16561 dataset, and key genes were screened through protein–protein interaction network analysis, machine learning, expression validation, and diagnostic evaluation. Co-enrichment analysis of key genes and DEMs was then conducted, followed by immune infiltration analysis, molecular network construction, drug prediction, and RT-qPCR validation.

**Results:**

A total of 103 and 51 DEMs were identified in the positive and negative ion modes, respectively, and were mainly enriched in steroid hormone biosynthesis. Five key genes—ITGAM, TLR4, MMP9, STAT3, and TLR2—were identified as significantly dysregulated in AIS and showed good diagnostic performance. RT-qPCR confirmed increased expression of TLR4, MMP9, and TLR2 in AIS, whereas ITGAM and STAT3 showed inverse trends. Joint enrichment analysis indicated that key genes and DEMs were mainly involved in 30 pathways, including the HIF-1 and FoxO signaling pathways. Immune infiltration analysis showed that ITGAM had the strongest negative correlation with memory B cells (*p* < 0.01, *r* = −0.39). In the clinical metabolomics cohort, 10 AIS patients and 10 non-stroke controls were included. AIS patients were 70.0% male (7/10) with a mean age of 65.70 ± 6.48 years, while controls were 60.0% male (6/10) with a mean age of 66.64 ± 7.18 years. Blood samples were obtained within 24 h of stroke onset. Stroke subtypes included large artery atherosclerosis (*n* = 3), cardioembolism (*n* = 5), and small artery occlusion (*n* = 2). None of the patients underwent intravenous thrombolysis or mechanical thrombectomy, all received standard medical treatment for AIS. In addition, 28 candidate drugs, including paregoric and celecoxib, were predicted.

**Conclusion:**

This exploratory study identified candidate hub genes and metabolites associated with AIS and highlighted their related pathways, immune features, molecular networks, and candidate drugs. These findings provide preliminary insights into the molecular basis and potential therapeutic targets of AIS.

## 1 Introduction

Stroke is a severe central nervous system disorder caused by acute cerebrovascular injury. According to global statistics, stroke is the second leading cause of death worldwide (1), with approximately 17% of the population experiencing a stroke during their lifetime, and about 10% of these individuals dying from it. Stroke is generally classified into two major types: hemorrhagic and ischemic. Among these, AIS is the most prevalent, accounting for approximately 70% of all stroke cases (2). It can occur across all age groups, but exhibits higher mortality and disability rates in middle-aged and elderly populations (3). In recent years, there has been a growing trend of onset at younger ages (4). AIS is primarily caused by obstruction of cerebral arterial blood flow, resulting in local cerebral ischemia and hypoxia, Its clinical manifestations vary depending on the location, extent, and severity of the lesion, but they are typically characterized by sudden onset and rapid progression, which triggers a cascade of pathological events including impaired energy metabolism, inflammation, and apoptosis (5). Despite advances in thrombolysis, mechanical thrombectomy, and antiplatelet therapies, a substantial proportion of patients still suffer from poor outcomes due to reperfusion injury or immune dysfunction (6). Therefore, it is imperative to explore the molecular potential pathways underlying AIS to identify more effective diagnostic and therapeutic targets.

With the rapid advancement of omics technologies, transcriptomics and metabolomics are increasingly utilized in stroke research (7). Transcriptomics enables the global analysis of gene expression under different physiological or pathological conditions, providing insights into the molecular basis of disease progression. Metabolomics, on the other hand, captures dynamic changes in metabolites that reflect both physiological states and pathological processes (8). Integrative analysis of transcriptomic and metabolomic data offers a multi-layered view of regulatory networks, facilitating the identification of core genes and pathways closely associated with disease. Compared to single-omics approaches, multi-omics strategies provide higher accuracy and greater systems-level insight, enhancing biomarker discovery and supporting molecular subtyping, early diagnosis, and personalized treatment (9, 10). Thus, applying a combined transcriptomic and metabolomic approach in AIS research may facilitate the comprehensive identification of pathogenic drivers and elucidate their potential pathways of action (11).

This study was based on whole blood samples collected from patients with acute AIS and healthy controls, and integrated transcriptomic datasets obtained from the Gene Expression Omnibus (GEO) database. By combining metabolomic profiling, transcriptomic analysis, and a suite of bioinformatics approaches, we systematically identified differentially expressed genes and metabolites. Subsequently, we constructed protein–protein interaction (PPI) networks, molecular regulatory networks, and a nomogram model for risk prediction. The expression levels of key candidate genes were further validated using real-time quantitative PCR (RT-qPCR). Our findings comprehensively reveal the interplay between metabolic dysregulation and immune-inflammatory responses in AIS, and identify several promising biomarkers and potential therapeutic targets. This study provides important theoretical support and methodological insights for the early diagnosis, prognostic assessment, and precision therapy of AIS.

## 2 Materials and methods

### 2.1 Metabolomic sequencing analysis

**Figure 1:**
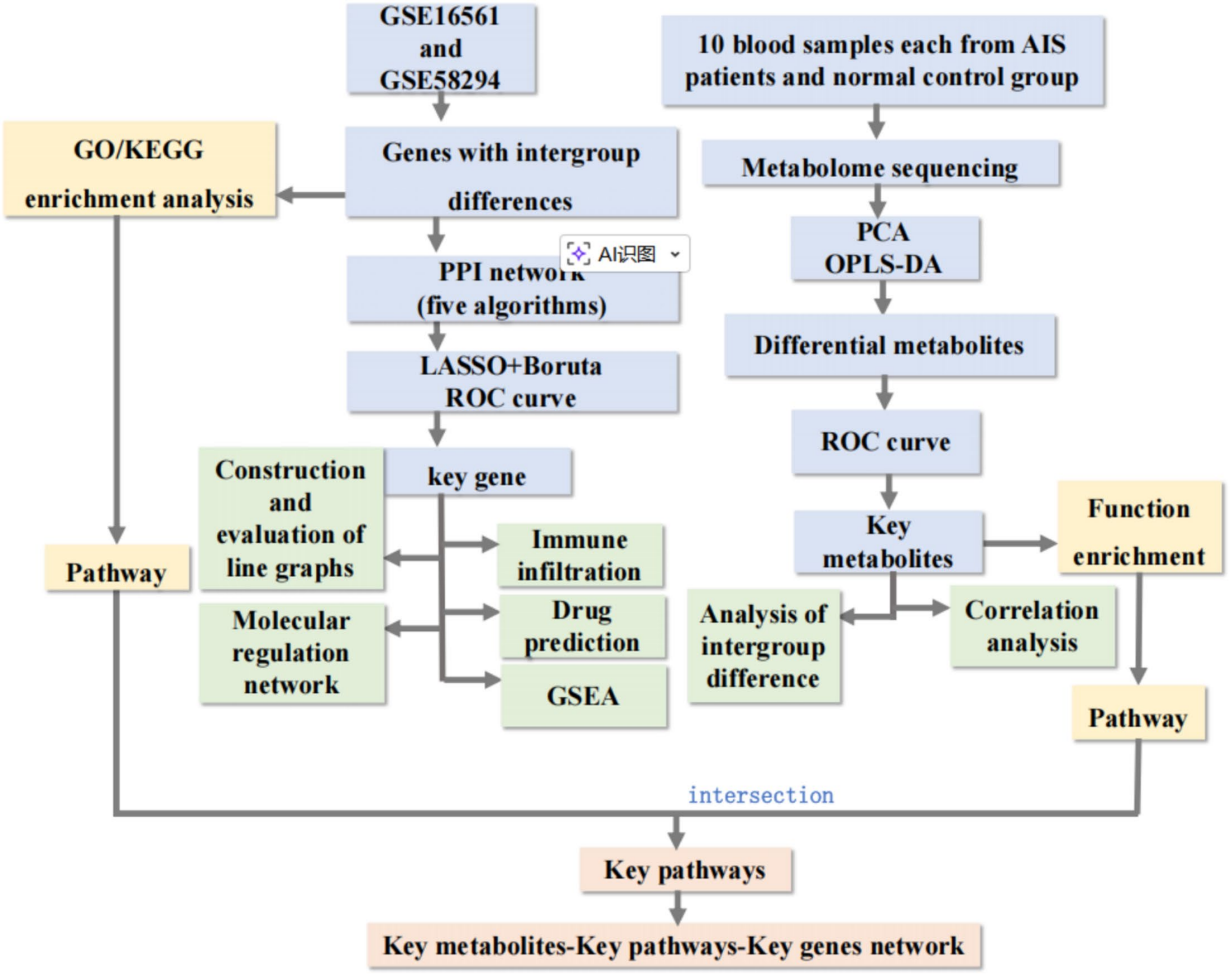
Schematic workflow of the study. This study integrated transcriptomic and metabolomic data to identify key biomarkers for acute ischemic stroke (AIS). Two data sources were used: (1) Clinical serum samples (10 AIS vs. 10 HC) for metabolome sequencing, quality control (QC), OPLS-DA analysis, and screening of differential expressed metabolites (DEMs) with KEGG enrichment; (2) GEO datasets (GSE16561 as training set, GSE58294 as validation set) for differential expressed genes (DEGs) screening, functional enrichment (GO/KEGG), PPI network construction, and key gene identification via machine learning (Boruta, SVM-RFE) and ROC curve validation. Subsequent analyses included immune infiltration, molecular regulatory network (TF-miRNA-lncRNA-mRNA) construction, drug prediction, and key gene-DEMs co-enrichment. Finally, key gene expression was validated by RT-qPCR using the same clinical samples.

The overall workflow of this study, integrating transcriptomic and metabolomic analyses to identify and validate key biomarkers for AIS, is systematically illustrated in Figure 1. Due to the pilot nature of the metabolomic profiling, this study was designed as an exploratory analysis to identify potential biomarkers that warrant further large-scale validation. The study was approved by the Ethics Committee of the Affiliated Hospital of Guizhou Medical University (Guizhou, China) (approval ID: 2019031 K) and was conducted in accordance with the principles of the Declaration of Helsinki. Written informed consent was obtained from all participants prior to enrollment. Whole blood samples were collected from 10 patients with acute AIS and 10 healthy controls (HC). The clinical characteristics of subjects in both groups are presented in Supplementary Table S1. Venous blood samples were collected using vacuum tubes without anticoagulants and allowed to clot at room temperature for 30 min to 1 h. After complete coagulation, the samples were centrifuged at 3,000 rpm for 15 min, and the resulting supernatant (serum) was carefully collected using a pipette or pipette tip, then stored at −80 °C. All participants were aged ≥18 years, and individuals with a history of cancer or neurodegenerative diseases were excluded from the study. Chromatographic separation was performed on an ACQUITY UPLC BEH C18 column (2.1 × 100 mm, 1.7 μm). The mobile phase consisted of water with 0.1% formic acid (A) and acetonitrile (B). The elution gradient was set as follows: 0–2 min, 5% B; 2–10 min, 5–95% B; 10–12 min, 95% B; 12–15 min, 5% B. The injection volume was 2 μL. Raw mass spectrometry data were processed using MS-DIAL for peak picking, alignment, and normalization.

Metabolomic profiling was performed using an ultra-performance liquid chromatography coupled with quadrupole time-of-flight mass spectrometry (UPLC-QTOF-MS) platform. The electrospray ionization (ESI) source operated in both positive and negative ion modes, with the following parameters: source temperature at 120 °C, desolvation temperature at 500 °C, desolvation gas (N₂) flow rate of 600 L/h, and cone gas (N₂) flow rate of 50 L/h. The capillary voltages were set at 3.0 kV (positive mode) and 4.5 kV (negative mode), with a sampling cone voltage of 27 eV and an extraction cone voltage of 4 eV. The quadrupole mass analyzer was operated in full scan mode over an m/z range of 50–1,500.

### 2.2 Quality control analysis

The reliability and reproducibility of the data were evaluated by total ion chromatogram (TIC). The stability of the data was assessed by looking at the coefficient of variation (CV) value distribution plots for all samples. In addition, the correlation between samples after quality control was estimated using Pearson’s to get an idea of the stability of the overall assay process.

### 2.3 Identification and enrichment analysis of differential expression metabolites

We executed Orthogonal Partial Least Squares Discriminant Analysis (OPLS-DA) analyses using Online Tools Meta-Analyser 6.0 and rejected outlier samples, followed by the construction of OPLS-DA model, while combining multivariate (variable importance in projection (VIP) ≥ 1) and univariate analyses. To control the false discovery rate (FDR), *p*-values were adjusted using the Benjamini-Hochberg method. Metabolites with an adjusted *p*-value < 0.05 and |log2(FC)| ≥ 1 were screened as differential expression metabolites (DEMs) to screen for DEMs between different samples (Two group comparison: Student’s *t* test; multiple group comparison: ANOVA). Further, KEGG analysis of DEMs was performed by MetaboAnalyst 6.0 (*p* < 0.05).

### 2.4 Data sources

GSE16561 and GSE58294, related to acute AIS, were extracted from the GEO database.^1^ The sequencing data of GSE16561, containing blood samples from 24 HC and 39 AIS patients, was acquired depending on GPL6883, and was considered as the training set. The sequencing data of GSE58294 was gained according to GPL570, which contained 92 blood samples (23 HC and 69 AIS) and was regarded as a validation set.

Data pre-processing involved several steps: (1) Probes without gene annotations were excluded. (2) For multiple probes mapping to a single gene, the avereps function was used to calculate the mean expression. (3) Missing values were imputed as 0. (4) Gene expression values were Z-score normalized (mean = 0, SD = 1) to ensure comparability across individual samples. Notably, to mitigate batch effects, the training set (GSE16561) and validation set (GSE58294) were analyzed independently rather than combined, ensuring the robustness and generalizability of the diagnostic model.

### 2.5 Differential expression and enrichment analysis

The differentially expressed genes (DEGs) were gained between HC and AIS samples via limma (v3.57.11) (12) in the training set (|log_2_FC| > 0.5, adjusted *p-*value < 0.05). Then, to probe pathways and functions related to DEGs, clusterProfiler (v4.9.4) (13) was applied to implement enrichment analysis for DEGs, including GO and KEGG (*p* value < 0.05).

### 2.6 Established protein–protein interaction network

To investigate the interactions between the corresponding proteins of DEGs, DEGs were included in the STRING database^2^ to structure the PPI network (Interaction score > 0.9), and that network was visualized using Cytoscape (v3.7.1) (14). Then, based on the genes with important roles in the PPI network, the top 50 genes were gained using Degree, Bottleneck, Eccentricity, Closeness, Betweenness, and Stress, respectively, and the common genes were selected further as the intersection genes for the subsequent analysis.

### 2.7 Filtering of key genes and construction of a nomogram

Based on the intersection genes, Boruta analysis was undertaken using Boruta (v8.0.0) (15) to filter the feature genes based on the importance ranking. Then, SVM-RFE analysis using e1071 (v1.7-14^3^) was performed to filter the feature genes. For SVM-RFE, we implemented 10-fold cross-validation (CV) to determine the optimal number of feature genes and to minimize the risk of overfitting. The accuracy and error rates across the CV folds were used to select the most stable feature subset. For the Boruta algorithm, we performed iterations to compare the importance of original features against shadow features, ensuring that only consistently significant ‘confirmed’ genes were carried forward. Next, feature genes common to both analyses were selected as candidate genes. Further, the expression profiles of these genes were extracted in the training and validation sets, and the genes with consistent expression trends in both datasets and marked differences between HC and AIS were selected as candidate key genes. Additionally, pROC (v1.18.4) (16) was employed to plot ROC curves of candidate biomarkers; genes with AUC values greater than 0.8 were considered as key genes for AIS. Moreover, to predict the risk of developing AIS, a nomogram containing key genes was built by rms (v6.7–1) (17). By calculating the score for each gene, a total score was obtained; the higher the total score, the higher the probability of developing AIS. Further, the predictive performance of the nomogram was evaluated by calibration curves and decision curves.

To ensure the reproducibility of our machine learning models, specific random seeds and parameters were applied. For Boruta feature selection, we set seed = 10, *p* = 0.01, and maxRuns = 50. SVM-RFE was executed with rfFuncs (random forest functions) and 10-fold cross-validation (seed = 96). Lasso regression and DCA were performed with seed = 3 and seed = 123, respectively.

### 2.8 Pathway enrichment analysis

To probe the pathways in which key genes were involved in the AIS process, gene set enrichment analysis (GSEA) was implemented by GseaVis (v0.0.8) (18). Firstly, correlation coefficients between each key gene and other genes were calculated by Spearman and ranked from high to low. The c2.kegg.symbols.gmt obtained from MSigDB was taken as the background gene set to perform GSEA (*p* < 0.05). We selected the top5 pathways for presentation.

### 2.9 Immune infiltration analysis

To assess the differences in immune status during the development of AIS, firstly, the proportion of 28 immune cells infiltrated in the different samples was calculated using ssGSEA using GSVA (v1.49.8) (19). The levels of immune cell infiltration between the AIS and HC groups were compared by Wilcoxon (*p* < 0.05). Next, the correlation between key genes and differential immune cells was explored by Spearman.

### 2.10 Molecular networks and drug prediction

To clarify whether the key genes were regulated by transcription factors (TF) and non-coding RNAs, firstly, the TFs related to the key genes were predicted in the TRRUST database, and the TF-mRNA network was structured. Then, miRNAs corresponding to biomarkers were predicted in miRWalk and miRDB databases, respectively, and those common to both databases were selected as target miRNAs. Subsequently, lncRNAs corresponding to target miRNAs were retrieved in the starbase database according to geneType = lincRNA and clipExpNum > 5, and in the miRNet database. Those predicted in both databases were selected as target lncRNAs. Then, the lncRNA-miRNA-mRNA network was established. Moreover, key gene-related drugs were forecasted in DGIdb, and a key gene-drug network was built.

### 2.11 Construction of DEMs-co-enrichment pathway-key gene map

KEGG co-enrichment analysis of key genes and DEMs was implemented by MetaboAnalyst 6.0. Further, the co-enrichment pathway, DEMs, and key genes were projected onto KEGG pathway maps using Pathview (v1.44.0) (20) to appreciate the relevant roles of DEMs and key genes in the co-enrichment pathway.

To quantitatively evaluate the relationship between the identified hub genes and key metabolites, a logFC product-based association analysis was performed (logFC_product_ = logFC_gene_ × logFC_metabolite_). We selected the top 10 DEMs from both positive and negative ion modes based on their AUC values. The correlation was quantified by calculating the product of the log_2_(Fold Change) of each gene.

### 2.12 Expression analysis of key genes

The expression levels of key genes from clinical samples were quantified by reverse transcription quantitative polymerase chain reaction (RT-qPCR). To ensure the independence and reliability of the validation, RT-qPCR was conducted in an independent validation cohort comprising 10 patients with AIS and 10 HC, who were recruited from March 2024 to June 2024 at the Affiliated Hospital of Guizhou Medical University. This cohort was independent of the samples used for the metabolomics analysis. Initially, TRIzol reagent (Ambion, Austin, United States) was utilized to extract total RNA from AIS blood samples and controls, followed by determination of the RNA concentration on NanoDrop (NanoPhotometer N50, Implen, Germany). Subsequently, cDNA was synthesized by reverse transcribing total RNA using the SureScript First-Strand cDNA synthesis kit (Servicebio, Wuhan, China). Next, quantitative analysis of gene levels was conducted using Universal Blue SYBR Green qPCR Master Mix (Servicebio, Wuhan, China). For quantitative analysis, the thermocycling conditions included 40 cycles with the following steps: pre-denaturation at 95 °C for 1 min, denaturation at 95 °C for 20 s, annealing at 55 °C for 20 s, and extension at 72 °C for 30 s. And the relative expression levels of the genes were assessed through the utilization of the 2^–△△Ct^ method. Besides, the primer sequences for the relevant genes were included in Supplementary Table S2.

### 2.13 Statistical analysis

All analyses were carried out using the R package (v4.3.1). For high-throughput data analysis (transcriptomics and metabolomics), multiple testing correction was performed using the Benjamini-Hochberg method, and FDR < 0.05 was considered statistically significant. For other experiments (e.g., RT-qPCR), a *p*-value < 0.05 was regarded as statistically meaningful.

## 3 Results

### 3.1 High stability and quality of metabolome sequencing data

**Figure 2:**
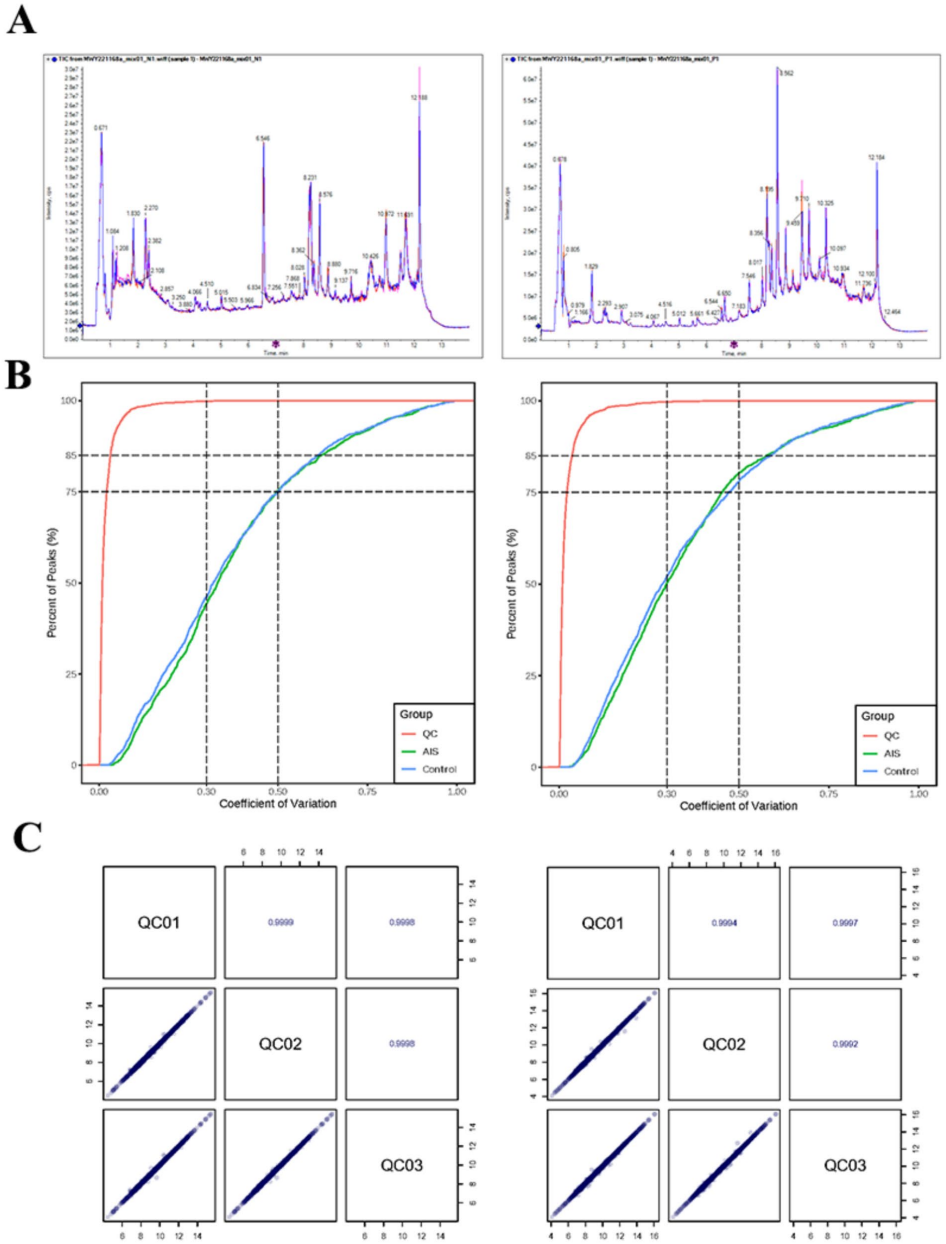
Results of metabolomic analysis. **(A)** Total ion chromatogram (TIC) overlay of quality control (QC) samples obtained from mass spectrometry analysis. The left panel represents the negative ion mode, and the right panel represents the positive ion mode. **(B)** Distribution of the coefficient of variation (CV) for each group of samples. The left panel shows data from the negative ion mode, and the right panel shows data from the positive ion mode. **(C)** Correlation analysis of QC samples. Diagonal cells indicate the QC sample names; cells below the diagonal display scatter plots of pairwise correlations between QC samples. The x- and y-axes represent the log-transformed metabolite abundance, and each dot corresponds to an individual metabolite.

The TIC plot revealed that the retention time and peak intensity of mass spectrometry detection of different QC samples were consistent, indicating better signal stability of mass spectrometry detection (Figure 2A). Moreover, the percentage of substances with a CV of less than 0.5 in the QC samples was higher than 85%, indicating that the experimental data were more stable (Figure 2B). In addition, the correlation between the samples was above 0.9, indicating the high quality of this data. Taken together, this indicated that the data could be applied to subsequent analyses (Figure 2C).

### 3.2 The 103 and 51 DEMs participated in multiple pathways

**Figure 3:**
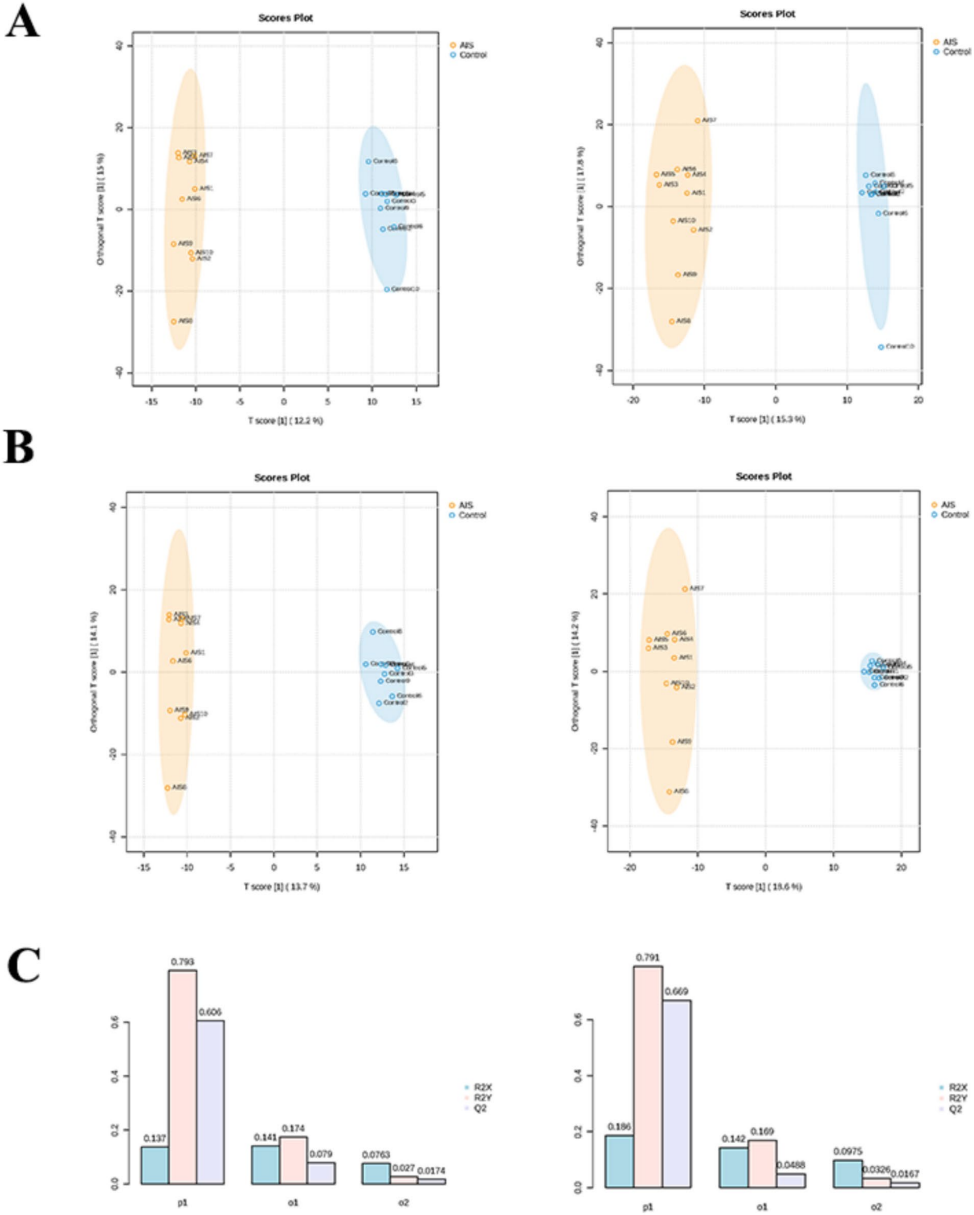
Orthogonal partial least squares discriminant analysis (OPLS-DA) for intergroup classification and model validation. **(A)** OPLS-DA score plots before outlier removal. The left panel represents the negative ion mode, and the right panel represents the positive ion mode. **(B)** OPLS-DA score plots after outlier removal. The left panel represents the negative ion mode, and the right panel represents the positive ion mode. **(C)** OPLS-DA permutation test plots after outlier removal. The left panel shows the negative ion mode, and the right panel shows the positive ion mode.

**Figure 4:**
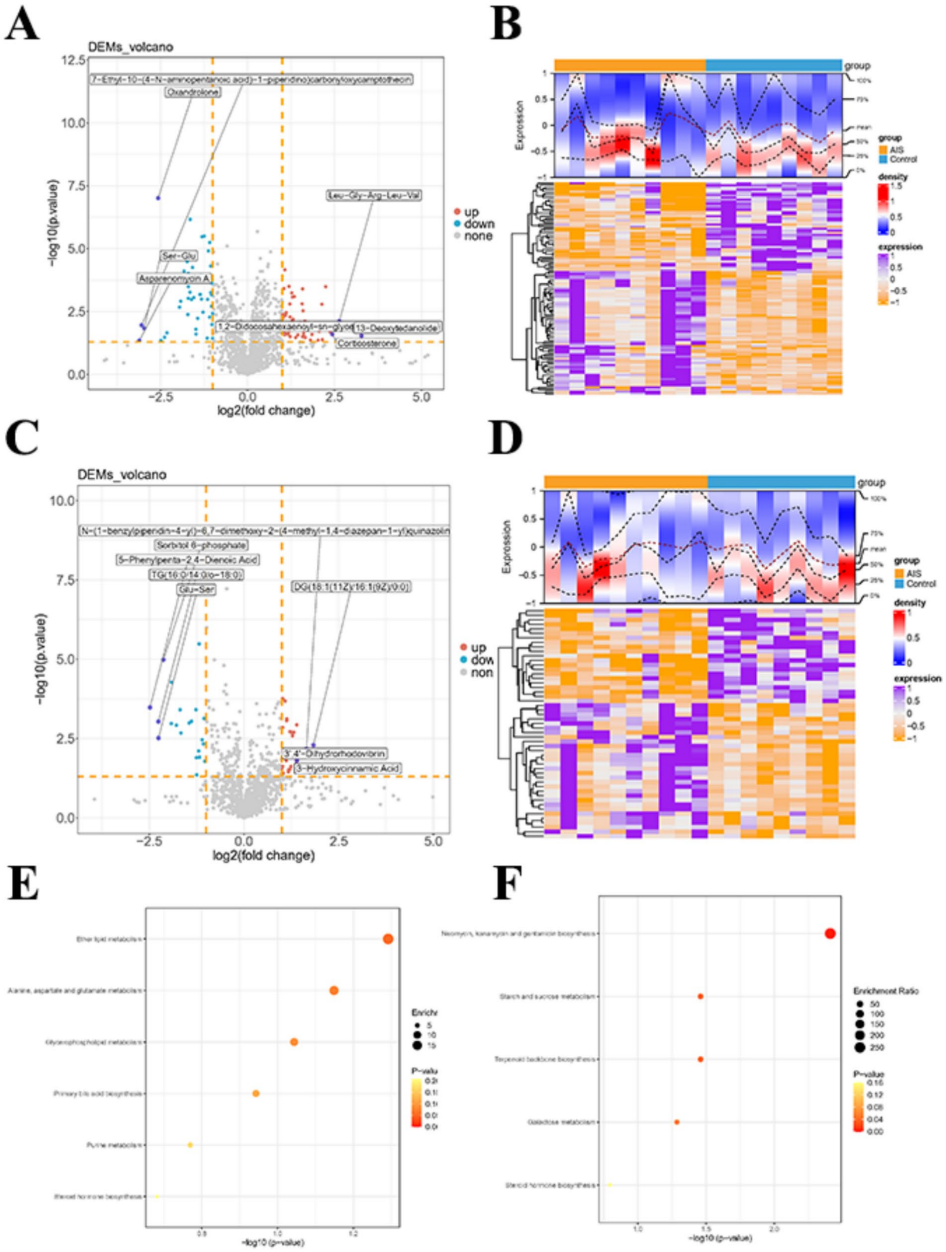
Identification and enrichment analysis of differential metabolites. **(A)** Volcano plot of differential metabolites in the negative ion mode. Each dot represents a metabolite: orange indicates upregulated metabolites, blue indicates downregulated metabolites, and gray indicates non-significant metabolites. **(B)** Heatmap of differential metabolites in the negative ion mode. Higher expression levels are shown in purple, and lower expression levels in orange. **(C)** Volcano plot of differential metabolites in the positive ion mode. Orange indicates upregulated metabolites, blue indicates downregulated metabolites, and gray indicates non-significant metabolites. **(D)** Heatmap of differential metabolites in the positive ion mode. Higher expression levels are shown in purple, and lower expression levels in orange. (E, F) KEGG enrichment bubble plots of differential metabolites. Panel **(E)** represents the positive ion mode, and panel **(F)** the negative ion mode. The x-axis shows –log10(adj.*p*-value), and the *y*-axis indicates the enriched pathways. The size of the bubbles reflects the enrichment ratio, and the color indicates the *p*-value.

The control 10 sample was found to be outliers by OPLS-DA analysis in the positive ion mode and was excluded. Then, the construction of the OPLS-DA model was found to separate the two groups well, and the R2Y and Q2Y were both above 0.6 (positive ion model: Q2 = 0.669, R2 Y = 0.791; negative ion model: Q2 = 0.606, R2 Y = 0.793), indicating that the model was reliable and predictive (Figures 3A–C). To mitigate the risk of over-fitting associated with the small sample size, we performed permutation tests (*n* = 200), and the results (Q2 intercept < 0) confirmed the high stability and statistical significance of the OPLS-DA model. Further, the 103 (60 up-regulated and 43 down-regulated) and 51 (30 up-regulated and 21 down-regulated) DEMs were derived between control and AIS in positive and negative ion modes, respectively (Figures 4A–D). Then, enrichment analysis of these DEMs revealed that these metabolites were associated with steroid hormone biosynthesis in both positive and negative ion modes (Figures 4E,F).

### 3.3 The 574 DEGs related to various pathways and functions

**Figure 5:**
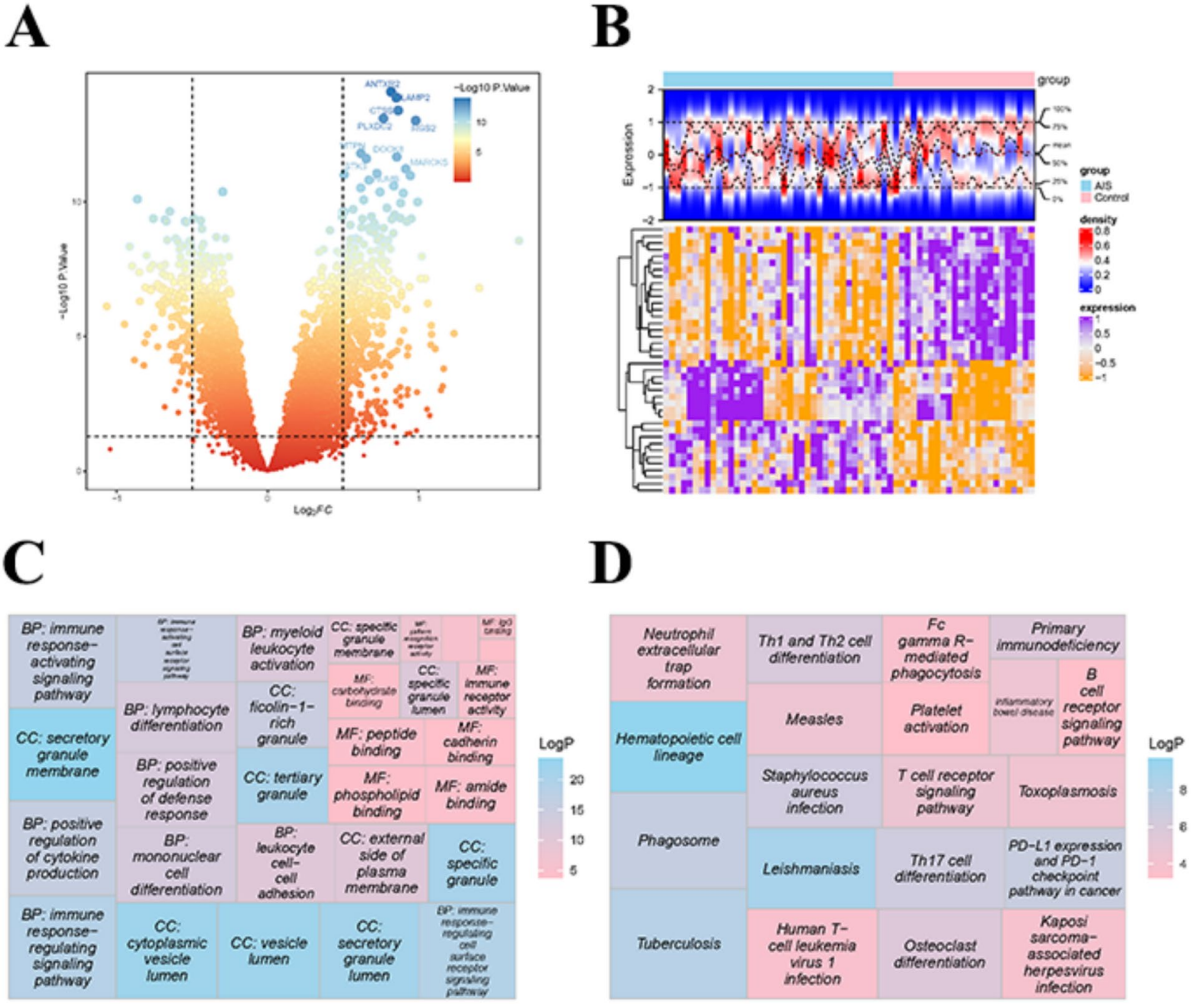
Differential expression analysis and functional enrichment results. **(A)** Volcano plot showing differentially expressed genes (DEGs) in the training set. Upregulated and downregulated genes are distinguished by color and position. **(B)** Heatmap displaying the expression levels of DEGs across individual samples. Color intensity represents the magnitude of gene expression. **(C)** KEGG pathway enrichment analysis of DEGs. The color gradient indicates the *p*-value, and the bubble size corresponds to the number of genes enriched in each pathway. **(D)** GO enrichment analysis of DEGs. Significantly enriched biological functions are presented, with color indicating statistical significance (*p*-value) and bubble size representing gene count.

The 574 DEGs were gained between HC and AIS samples, which included 450 up-regulated and 124 down-regulated genes (Figures 5A,B). Then, GO items indicated that these genes were primarily related to immune response-activating signaling pathway, positive regulation of cytokine production, immune response-activating cell surface receptor signaling pathway, etc. (Figure 5C). KEGG pathway showed that they were mainly involved in neutrophil extracellular trap formation, PD-L1 expression, and PD-1 checkpoint pathway in cancer, B cell receptor signaling pathway, T cell receptor signaling pathway, etc. (Figure 5D).

### 3.4 ITGAM, TLR4, MMP9, STAT3, and TLR2 regarded as key genes

**Figure 6:**
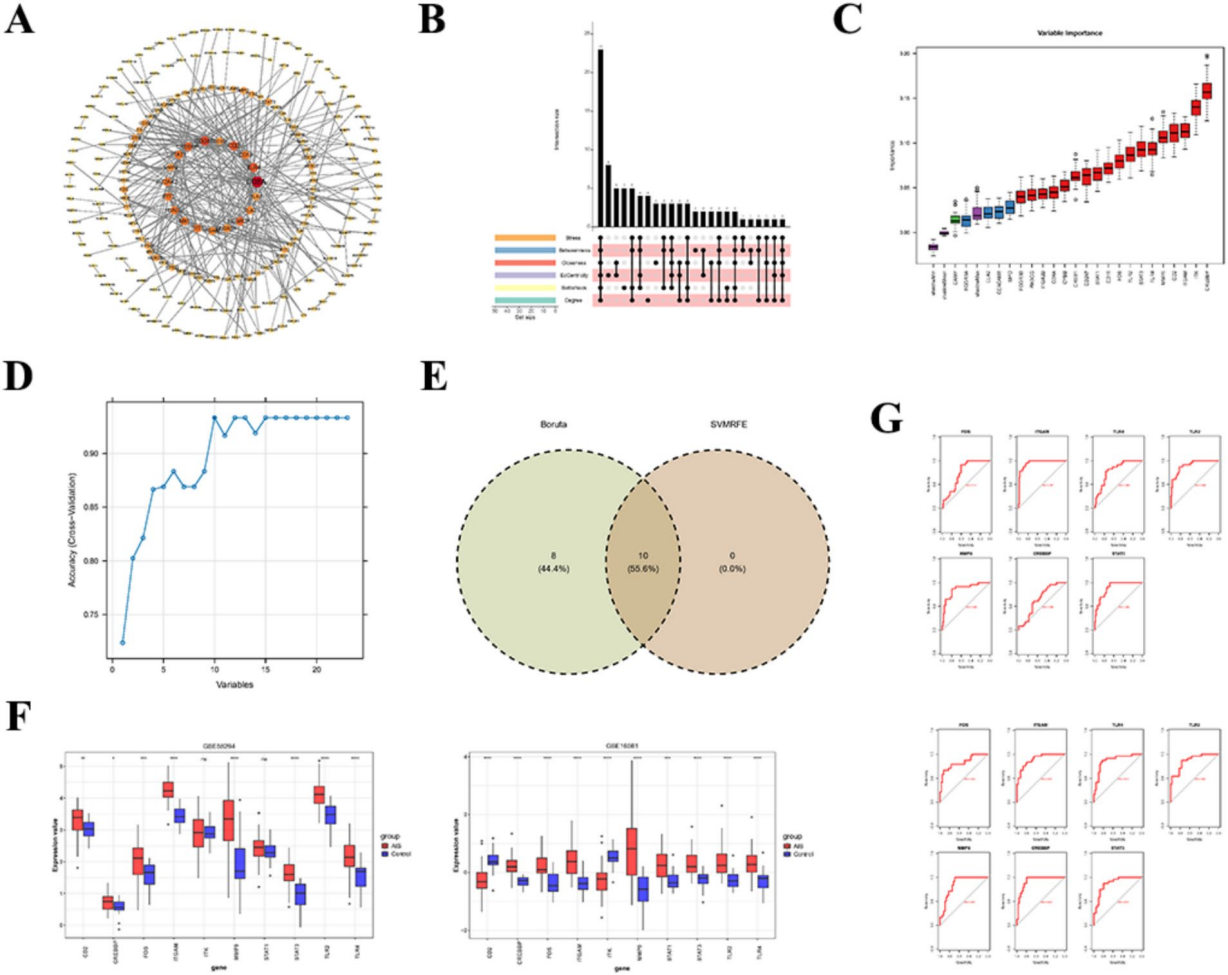
Identification of key genes and evaluation of diagnostic performance. **(A)** Protein–protein interaction (PPI) network analysis. The color and size of the nodes represent the degree of gene interaction. **(B)** Candidate genes were selected as the intersection of the top 50 genes ranked by six different topological metrics. **(C)** Boruta feature selection results. The purple box plots represent the minimum, average, and maximum *Z*-scores of shadow attributes; green box plots indicate rejected attributes; blue box plots indicate tentative attributes; and red box plots indicate confirmed attributes. **(D)** Support vector machine–recursive feature elimination (SVM-RFE) analysis results. **(E)** Venn diagram showing the intersection of genes selected by Boruta and SVM-RFE analyses, defined as key genes. **(F)** Expression patterns of the key genes in the training and validation sets. Left: validation set; right: training set. **(G)** Receiver operating characteristic (ROC) curves of key genes in the training and validation sets.

Isolated genes were eliminated based on an interaction score >0.9, and a PPI network containing 331 points and 329 edges was established, in which CD8A, CD247, and CREBBP had strong interactions with other genes (Figure 6A). Further, among the TOP50 genes screened by each of the 6 topological metrics, 23 of them were common as intersection genes (Figure 6B). Then, 18 and 10 signature genes were acquired by Boruta (Figure 6C) and SVM-RFE (Figure 6D) analyses, respectively, with 10 common genes as candidate genes (Figure 6E), including CREBBP, ITK, ITGAM, CD2, TLR4, MMP9, STAT3, FOS, TLR2 and STAT1. Moreover, CREBBP, ITGAM, TLR4, MMP9, STAT3, FOS, and TLR2 were all remarkably overexpressed in the AIS group in the training set and were the same in the validation set (*p* < 0.05) (Figure 6F). In addition, except for FOS and CREBBP, the AUC values of the other five genes were all greater than 0.8, making ITGAM, TLR4, MMP9, STAT3, and TLR2 as key genes (Figure 6G).

### 3.5 Establishment of a nomogram that predicts the probability of developing AIS

**Figure 7:**
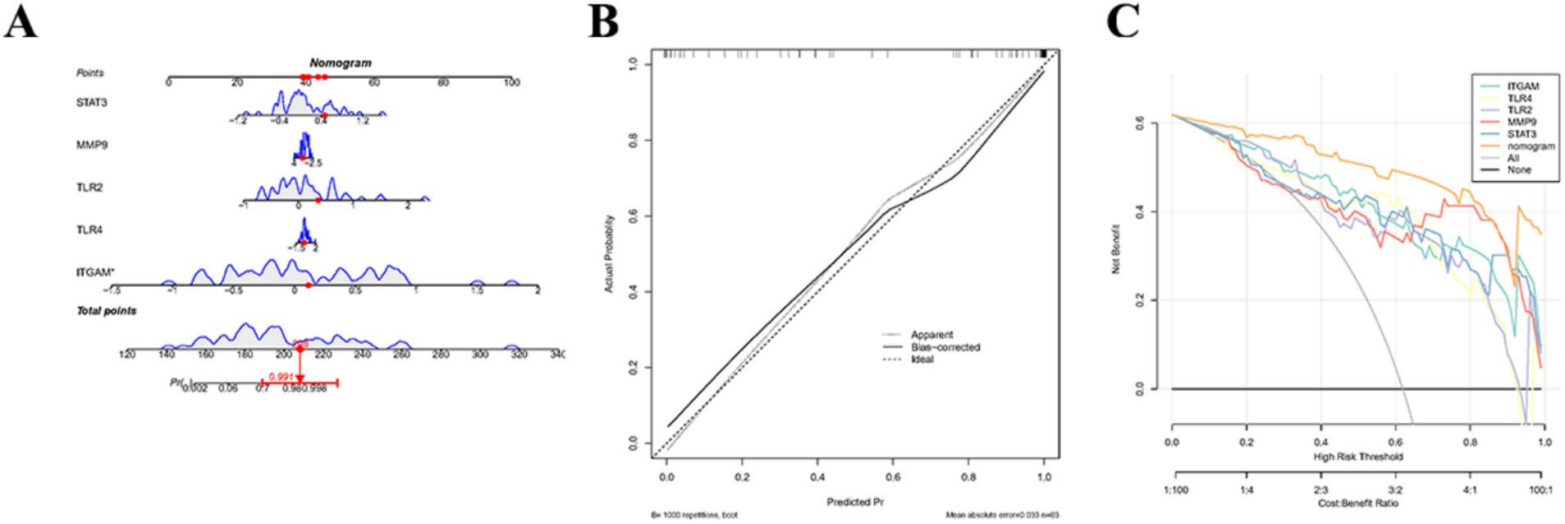
Construction and evaluation of the nomogram. **(A)** Nomogram based on key genes for diagnostic prediction. **(B,C)** Evaluation of the diagnostic model derived from the nomogram. Left panel Calibration curve, where the x-axis represents the predicted probability and the y-axis represents the actual observed probability. Right panel Decision curve analysis (DCA), where the x-axis represents the threshold probability and the y-axis represents the net benefit after subtracting harm from benefit. Curves in different colors correspond to different key genes.

Based on five key genes, a nomogram was constructed to predict the probability of developing AIS (Figure 7A). Then, the calibration curves largely coincided with the ideal curves (Figure 7B), and the net yields of the nomogram in the decision curves were higher than those of each factor (Figure 7C), which comprehensively indicated that the model had excellent predictive ability.

### 3.6 Key genes involved in multiple pathways in AIS

**Figure 8:**
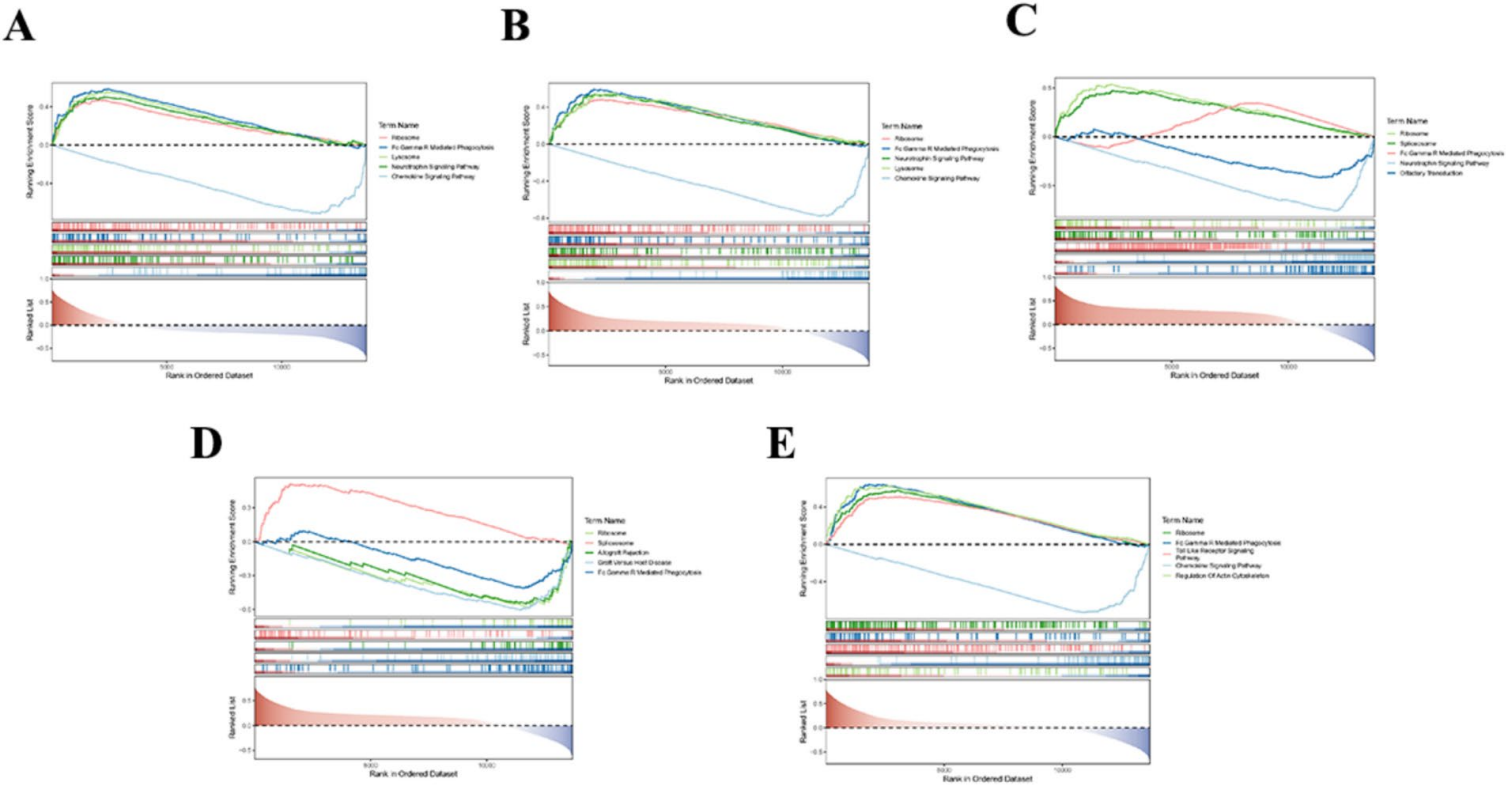
GSEA analysis results. Panels **(A–E)** represent gene set enrichment analysis (GSEA) results for ITGAM, MMP9, STAT3, TLR2, and TLR4, respectively.

We provided information about the possible pathways associated with key genes in AIS by GSEA. The 5 key genes were all associated with the ribosome, Fc gamma R-mediated phagocytosis. In addition, ITGAM, MMP9, and TLR4 were also remarkably linked to the chemokine signaling pathway. Moreover, ITGAM, MMP9, and STAT3 were also involved in the neurotrophin signaling pathway (Figures 8A–E). It also suggested that these genes might impact the onset and development of AIS through related pathways.

### 3.7 Key genes played a role in the immune microenvironment of AIS

**Figure 9:**
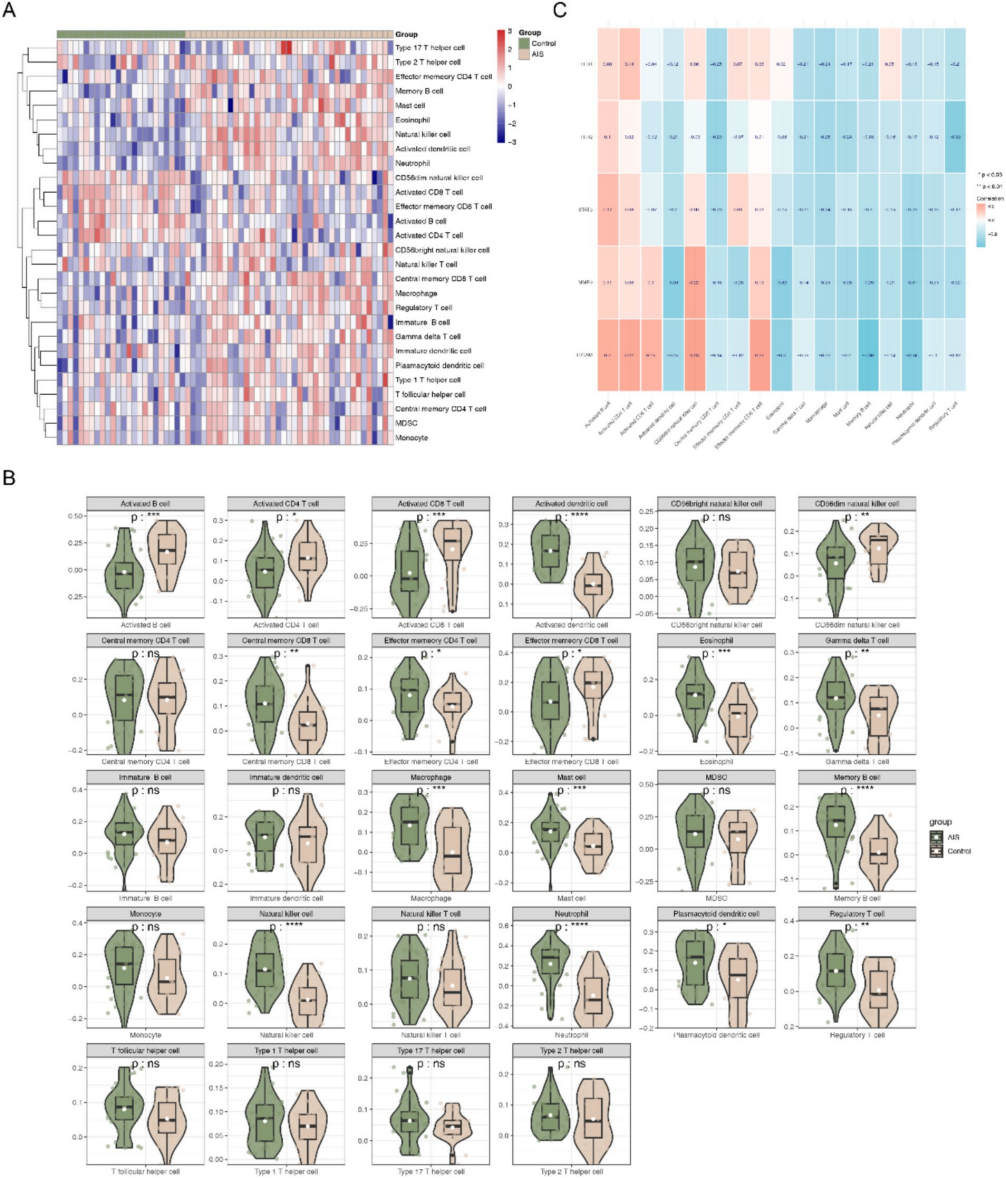
Analysis of immune infiltration results. **(A)** Heatmap of immune cell infiltration scores across samples calculated using the single-sample gene set enrichment analysis (ssGSEA) algorithm. **(B)** Box plot showing immune infiltration scores of different immune cell types across groups. **(C)** Correlation analysis between key genes and differentially infiltrated immune cell types.

The immune scores of 28 immune cells in different samples were probed (Figure 9A). Of these, 17 immune cells showed remarkable differences in their infiltration levels between the AIS and HC groups (*p* < 0.05). For example, activated B cell, activated CD4 T cell, activated CD8 T cell were remarkably higher in HC group, while activated dendritic cell, Macrophage, natural killer cell were notably higher in AIS group (Figure 9B). In addition, ITGAM had the strongest marked negative correlation with memory B cells (*p* < 0.01, cor = −0.39) (Figure 9C).

### 3.8 Discussions of key gene-related regulatory networks and drugs

**Figure 10:**
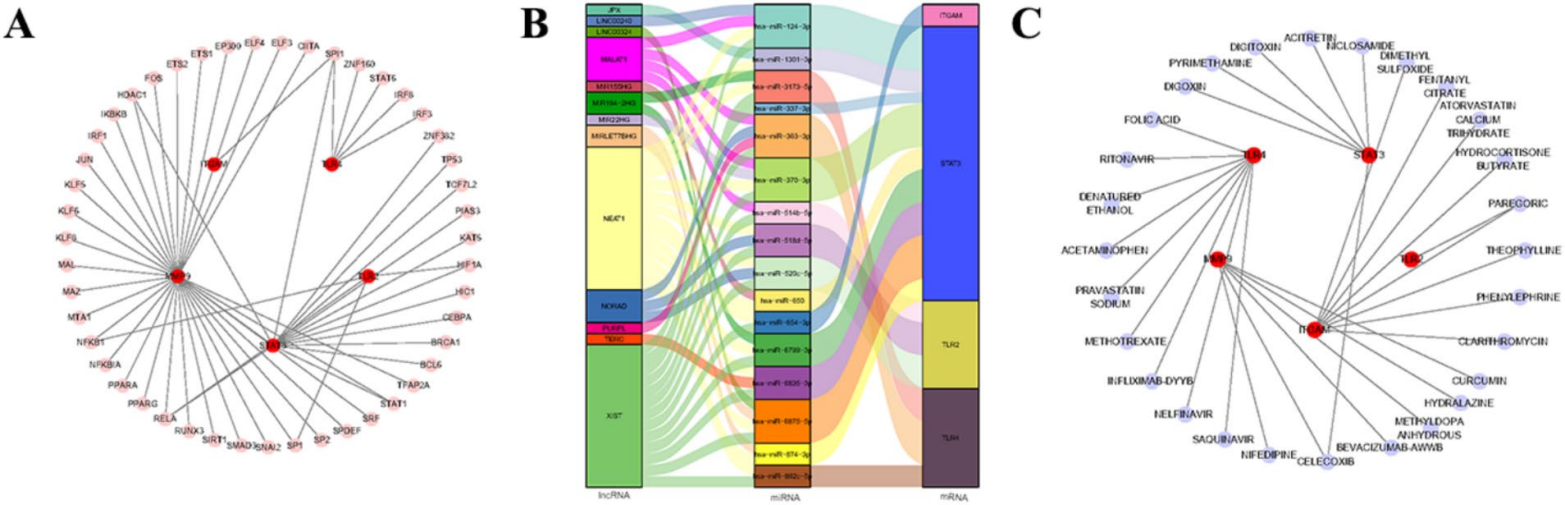
Regulatory network and drug prediction analysis of key genes. **(A)** Transcription factor (TF)–key gene regulatory network. Red nodes represent key genes, and pink nodes represent transcription factors. **(B)** CeRNA Sankey diagram illustrating the competing endogenous RNA regulatory relationships. **(C)** Drug–key gene interaction network. Red nodes indicate key genes, and purple nodes represent predicted drug compounds.

A total of 47 TF were predicted in the TRRUST database; among them, ITGAM, TLR4, and STAT3 were all regulated by SPI1, and MMP9 and TLR2 were both regulated by NFKB1 and SP1 (Figure 10A). Then, 72 target miRNAs were acquired in miRWalk and miRDB databases. Next, 44 miRNA-lncRNA pairs were derived from the starbase database and the miRNet database. Subsequently, the lncRNA-miRNA-mRNA network was constructed, such as XIST-hsa-miR-370-3p-STAT3, NEAT1-hsa-miR-124-3p-STAT3 (Figure 10B). Further, the 28 drugs associated with key genes were predicted in the DGIdb database. Among them, both TLR2 and ITGAM predicted paregoric, while both MMP9 and STAT3 predicted celecoxib (Figure 10C). These computationally predicted drugs may serve as potential candidates for AIS therapy; however, their actual efficacy and safety require further rigorous experimental and clinical validation.

### 3.9 Key genes and DEMs are dramatically enriched in 30 pathways

**Figure 11:**
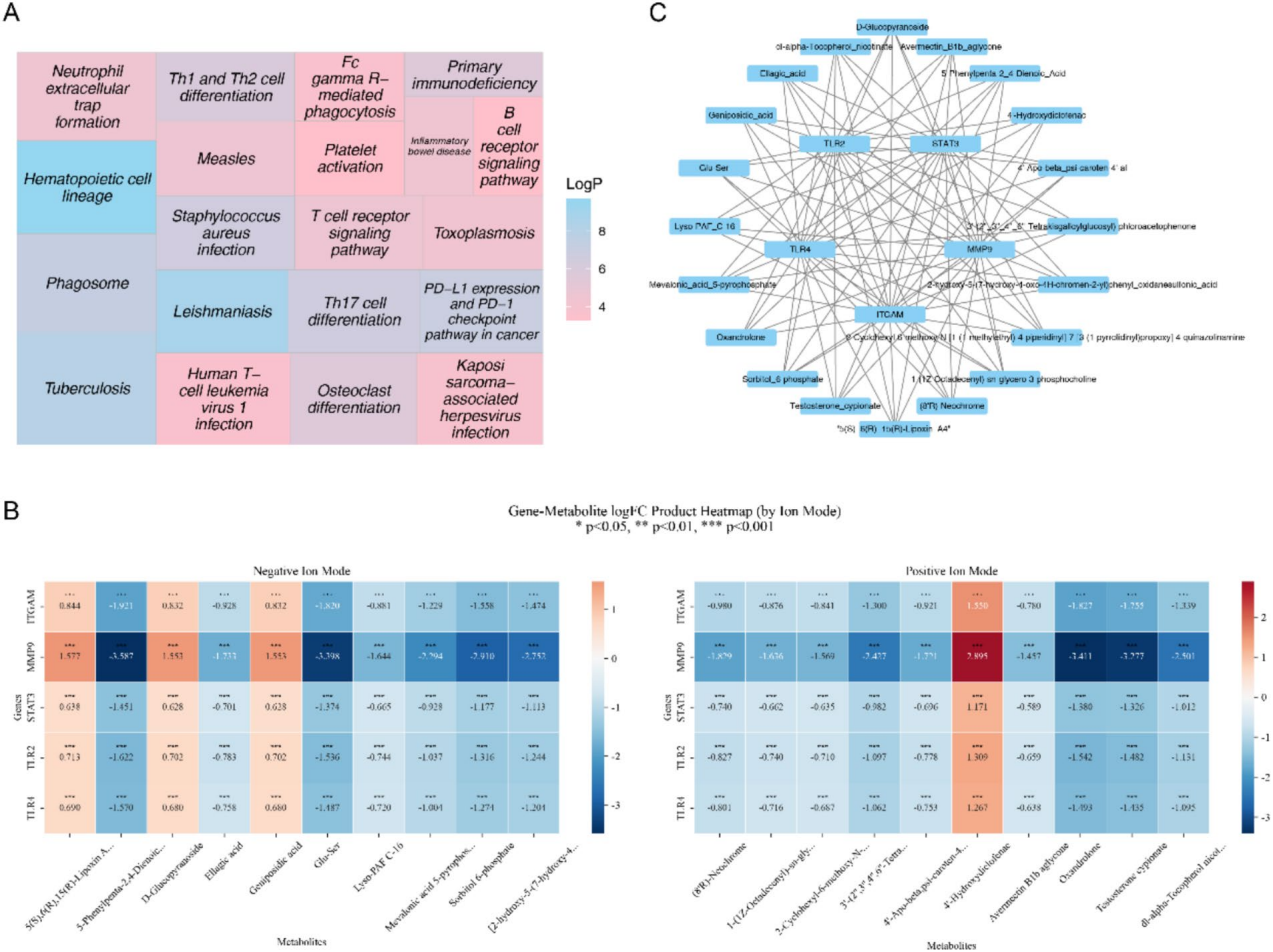
Quantitative multi-omics integration. **(A)** KEGG enrichment analysis. **(B)** Heatmap of logFC product between 5 hub genes and top 20 metabolites. Red cells (logFC_product_ > 0) indicate concordant changes; blue cells (logFC_product_ < 0) indicate discordant changes. Numerical values indicate the magnitude of the association. **(C)** Gene-metabolite association network visualized by Cytoscape, where nodes represent genes and metabolites, and edges reflect their quantitative links.

We explored pathways where key genes were co-associated with DEMs, helping to provide new insights into the treatment of AIS. Key genes and DEMs were enriched in 30 notable pathways (*p* < 0.05), like Hepatitis B, PD-L1 expression, and PD-1 checkpoint pathway in cancer, HIF-1 signaling pathway and FoxO signaling pathway, Toll-like receptor signaling pathway, etc. These pathways might play a potential role in the diagnostic and therapeutic process of AIS (Figure 11A). In addition, Figure 11A demonstrated the relationship between DEMs and key genes in the co-enrichment pathway.

Beyond pathway enrichment, we performed a quantitative association analysis to reveal the synergy between candidate genes and metabolites (Figure 11B). TLR4 showed strong concordant associations (red) with several reparative metabolites, particularly in the negative ion mode with Lipoxin A4 (logFCproduct = +0.850) and Mevalonic acid 5-pyrophosphate (logFC product = +0.729), suggesting a coordinated metabolic response linked to inflammation resolution. MMP9 exhibited the most dramatic fluctuations, showing a strong concordant relationship with the synthetic androgen Oxandrolone (logFCproduct = +2.895) in positive mode, while displaying discordant changes (blue) with Avermectin B1b aglycone (logFCproduct = −3.411). This inverse relationship suggests that certain exogenous compounds might influence MMP9 activity during stroke progression.

The pro-inflammatory cluster (ITGAM, TLR2, and STAT3) showed highly similar patterns, predominantly exhibiting discordant changes with endogenous repair-related metabolites like Geniposidic acid and Ellagic acid in the negative ion mode. To further visualize these interactions, a multi-omics network was constructed, highlighting the dense regulatory hub formed by the 5 key genes and 20 core metabolites (Figure 11C).

### 3.10 Validation of the expression of key genes in clinical samples

**Figure 12:**
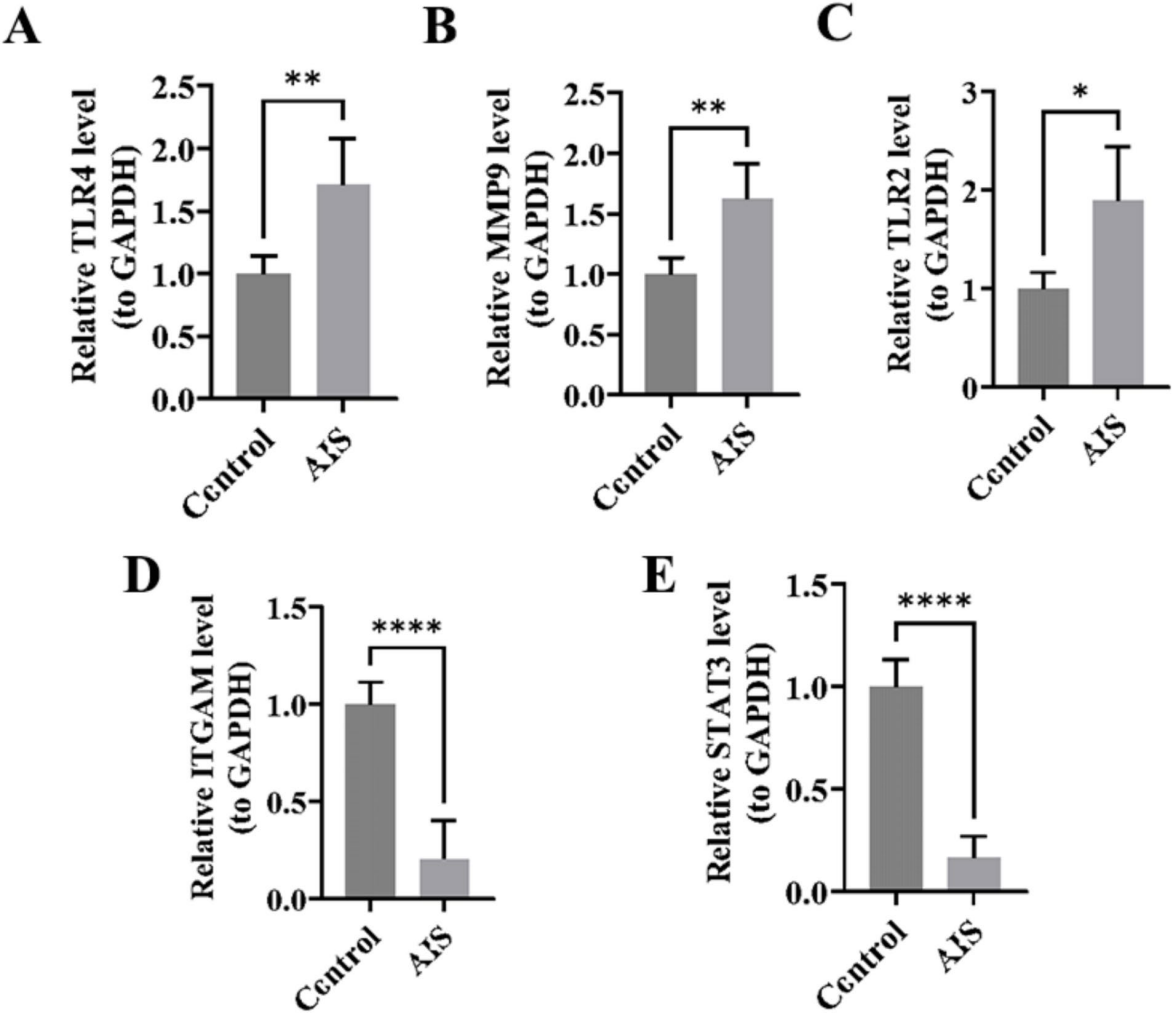
PCR validation results. Panels **(A–E)** show the PCR results for TLR4, MMP9, TLR2, ITGAM, and STAT3, respectively.

Consistent with the results mentioned above, the key genes TLR4, MMP9, and TLR2 exhibited significantly elevated expression levels in AIS samples when compared to control samples (Figures 12A–C). Nonetheless, a contrasting pattern was observed in the expression levels of the genes ITGAM and STAT3, both showing significantly lower levels in AIS samples compared to control samples, which may be attributed to various complex factors (Figures 12D,E).

## 4 Discussion

Our multi-omics analysis provides a systematic investigation of the molecular potential pathways underlying acute AIS. AIS is a cerebrovascular disease characterized by high incidence and disability rates, with complex pathogenesis that remains incompletely understood (21), particularly in the areas of immune regulation and metabolic imbalance (22). Mitophagy has also been implicated in IS by regulating oxidative stress and energy metabolism (23). Traditional diagnostic methods suffer from limited sensitivity and poor early detection, emphasizing the need to identify novel molecular targets with diagnostic and therapeutic relevance. From the integrative transcriptomic and metabolomic analysis, we identified five key genes—ITGAM, TLR4, MMP9, STAT3, and TLR2—and multiple differential metabolites that were significantly enriched in 30 functionally relevant pathways, including the HIF-1 signaling pathway, FoxO signaling pathway, and TLR signaling pathway. Furthermore, immune infiltration analysis, regulatory network modeling, and drug prediction were combined to comprehensively elucidate the roles of these key genes in AIS, expanding our understanding of its molecular landscape and providing theoretical support for future diagnosis and therapeutic strategies. Although the metabolomic cohort was relatively small, the reliability of the identified DEMs was cross-validated through our integrated multi-omics approach. The key metabolites were significantly enriched in pathways (e.g., steroid hormone biosynthesis and HIF-1 signaling) that showed strong biological consistency with the DEGs identified from larger transcriptomic datasets (GSE16561 and GSE58294). In this study, we employed a rigorous machine learning pipeline to mitigate the risk of overfitting, which is a common challenge in transcriptomic studies with small sample sizes. By integrating two distinct feature selection algorithms (Boruta and SVM-RFE with 10-fold cross-validation) and, more importantly, validating the diagnostic efficacy in a completely independent dataset (GSE58294), we ensured the stability and reproducibility of the 5-gene signature. This multi-layered validation approach provides strong evidence that our results are biologically meaningful rather than artifacts of data overfitting. In this study, we employed a rigorous machine learning pipeline to mitigate the risk of overfitting, which is a common challenge in transcriptomic studies with small sample sizes. By integrating two distinct feature selection algorithms (Boruta and SVM-RFE with 10-fold cross-validation) and, more importantly, validating the diagnostic efficacy in a completely independent dataset (GSE58294), we ensured the stability and reproducibility of the 5-gene signature. This multi-layered validation approach provides strong evidence that our results are biologically meaningful rather than artifacts of data overfitting. This multi-layered alignment reduces the likelihood of false-positive findings stemming from a single-omics cohort.

TLR4 and TLR2 are classified within the same group, both belonging to the Toll-like receptor (TLR) family, which comprises transmembrane proteins capable of recognizing exogenous pathogen-associated molecular patterns (PAMPs) and endogenous damage-associated molecular patterns (DAMPs). These receptors play a critical role in the initiation of inflammatory responses in the innate immune system. Structurally, TLRs are characterized by large extracellular leucine-rich repeat domains and a conserved intracellular Toll/IL-1 receptor (TIR) domain. With the exception of TLR3, all members of the TLR family signal through the adaptor molecule MyD88 (24). In AIS, TLR4 and TLR2 exhibit distinct and even opposing biological roles. Compared to wild-type mice, TLR4 knockout mice demonstrate significantly smaller infarct volumes and lower mortality following cerebral ischemia/reperfusion injury. Mechanistically, TLR4 aggravates ischemic damage by activating the NF-κB signaling pathway. In contrast, TLR2 knockout mice show larger infarct volumes and higher mortality than their wild-type counterparts, suggesting that TLR2 may exert a protective role against ischemic injury. This protection is thought to be mediated by the activation of the PI3K/Akt and ERK1/2 signaling pathways (25). Moreover, clinical studies have shown that the expression levels of TLR2 and TLR4 at admission positively correlate with inflammatory cytokines (IL-1β, IL-6, TNF-α) and adhesion molecules (VCAM1) measured at 24 h, 72 h, and 7 days post-stroke. TLR4 is also positively associated with ICAM1 levels, indicating that both receptors contribute to AIS pathogenesis through the activation of inflammatory pathways (26). While our study focuses on the clinical identification and diagnostic performance of these receptors, their functional roles in AIS pathophysiology are well-supported by prior literature. For instance, functional studies using TLR4-knockout mice have demonstrated a significant reduction in infarct volume and improved neurological outcomes by inhibiting the NF-κB-mediated pro-inflammatory cascade (27). Similarly, the activation of TLR2 has been shown to modulate the early immune response, where its deficiency can alter the polarization of microglia, thereby affecting neuronal survival (28). These findings align with our observation of significant TLR4 and TLR2 upregulation in AIS patients, reinforcing their potential as both biomarkers and drivers of ischemic injury. In the present study, the expression levels of TLR4 and TLR2 were significantly upregulated in AIS samples compared to controls. This finding suggests that the upregulation of TLR4 and TLR2 may reflect the complex host response to ischemic injury, involving both pro-inflammatory damage and potential compensatory potential pathways.

ITGAM and MMP9 play important and interconnected roles in the pathophysiology of acute AIS, particularly through their involvement in inflammation and immune regulation. Both genes have been shown to be significantly overexpressed in stroke patients and demonstrate notable diagnostic potential according to ROC curve analysis (29). ITGAM (also known as CD11b) facilitates leukocyte adhesion to vascular endothelium and transendothelial migration, while MMP9 directly disrupts the blood–brain barrier (BBB) by degrading basement membrane components and tight junction proteins (29, 30). Pericytes also play an important role in blood–brain barrier integrity and neuroinflammation after ischemic stroke (31). During stroke progression, ITGAM modulates the secretion of key inflammatory cytokines such as TNF-α, IL-1β, and IL-6, and influences neuronal apoptosis. MMP9, primarily released by neutrophils, contributes to BBB breakdown, brain edema, and hemorrhagic transformation, indicating that both genes synergistically participate in the secondary injury cascade of AIS (29). Clinically, ITGAM and MMP9 not only serve as potential diagnostic biomarkers but also represent therapeutic targets. Notably, ITGAM inhibition may offer anti-inflammatory benefits, whereas the combination of MMP9 inhibitors with thrombolytic therapy may enhance the safety and efficacy of acute stroke treatment (32). In the present study, MMP9 expression was significantly elevated in AIS samples, whereas ITGAM exhibited an opposite trend. These findings suggest that ITGAM and MMP9 may exert distinct regulatory functions at different time points or pathological stages of AIS, reflecting the complexity of immune and vascular responses during stroke progression. The synergism between ITGAM and MMP9 is critical for BBB disruption. Prior *in vivo* evidence indicates that MMP9-neutralizing antibodies can stabilize the neurovascular unit by preventing the degradation of tight junction proteins like claudin-5 (32). Furthermore, ITGAM (CD11b) deficiency in animal models leads to reduced neutrophil infiltration into the brain parenchyma, confirming its essential role in transendothelial migration (30). Our results provide clinical validation for these established experimental potential pathways.

The most extensively studied function of STAT3 is its role as a survival factor, providing protection to various cell types against pro-apoptotic stimuli. In the rodent brain, STAT3 has been shown to exert neuroprotective effects in the context of stroke. Mice with endothelial-specific STAT3 deficiency exhibit significantly larger infarct volumes 24 h after middle cerebral artery occlusion (MCAO) compared to wild-type controls, suggesting that endothelial STAT3 contributes to tissue protection (33). However, excessive activation of STAT3 in microglia can exacerbate microglial activation and neuroinflammation following ischemic stroke. Inhibition of the JAK2/STAT3 pathway has been shown to alleviate cerebral ischemic injury and neuroinflammation by suppressing mitochondrial activity in microglia (34). Additionally, elevated levels of circulating miR-155, JAK2/STAT3, and TNF-α in patients with acute ischemic stroke may trigger post-stroke inflammatory cascades (35). Collectively, these findings suggest a dual role for STAT3 in acute ischemic stroke: while endothelial STAT3 appears to be protective, overactivation in microglia may contribute to detrimental inflammatory responses. The context-specific function of STAT3 underscores the complexity of its involvement in stroke pathophysiology and highlights the importance of cell-type–specific regulatory potential pathways in designing targeted therapies. Beyond its role as a survival factor, the functional activation of STAT3 in microglia has been shown to drive neuroinflammation. Studies utilizing microglia-specific STAT3 deletion demonstrated reduced secretion of TNF-α and IL-6 after middle cerebral artery occlusion (MCAO), highlighting its indispensable role in the inflammatory cascade (36).

Our study revealed that the immune microenvironment in the AIS group is characterized by significantly increased activation of dendritic cells, macrophages, and natural killer (NK) cells. Following AIS onset, the integrity of the blood–brain barrier is disrupted, allowing circulating monocytes to rapidly infiltrate the lesion site within 24 h and peak between 3 to 7 days post-stroke (30). These infiltrating monocytes subsequently differentiate into functionally specialized macrophages (17). In the early stages of disease, macrophages typically exhibit a pro-inflammatory phenotype, characterized by the activation of pattern recognition receptors such as Toll-like receptors, which promote the production of pro-inflammatory cytokines. This response exacerbates neuroinflammation, contributes to brain edema, and induces neuronal death (37). Notably, FOXP3^+^ macrophages have been identified in the peri-infarct regions of stroke-affected brains, where they positively regulate phagocytosis and the degradation of phagocytic cargo (38). These findings suggest that macrophages play a dual role in AIS, highlighting the therapeutic potential of precisely targeting their polarization and subpopulation dynamics.

Regulatory network analysis revealed that SPI1 may serve as a common transcriptional regulator of ITGAM, STAT3, and TLR4. Moreover, STAT3 was found to be modulated by multiple miRNAs (36) and incorporated into a complex ceRNA network involving lncRNAs such as NEAT1 (39), suggesting multi-level post-transcriptional regulation in AIS. We also predicted 28 candidate drugs that potentially target the identified genes. Notably, celecoxib, a selective COX-2 inhibitor and potential inhibitor of MMP9 and STAT3, has shown promise in post-stroke inflammation suppression (40). Other candidates, such as paregoric, require further experimental validation. In response to the need for quantitative integration, our strategy evolved from a pathway-centric approach (identifying shared biological functions) to a data-driven association method. By directly linking hub genes like TLR4 and MMP9 to specific metabolic shifts (such as steroid and lipid metabolism) via logFC product analysis, we provide a more granular understanding of how the immune-inflammatory response drives the metabolic landscape of AIS. This quantitative bridge between transcriptomics and metabolomics enhances the reliability of the identified biomarkers and reveals potential targets for metabolic intervention. In response to the need for quantitative integration, our strategy evolved from a pathway-centric approach (identifying shared biological functions) to a data-driven association method. By directly linking hub genes like TLR4 and MMP9 to specific metabolic shifts (such as steroid and lipid metabolism) via logFC product analysis, we provide a more granular understanding of how the immune-inflammatory response drives the metabolic landscape of AIS. This quantitative bridge between transcriptomics and metabolomics enhances the reliability of the identified biomarkers and reveals potential targets for metabolic intervention.

## 5 Conclusion and perspectives

In summary, this exploratory study identified five potential AIS-related hub genes and corresponding metabolites. We elucidated their potential roles in immune regulation, metabolic pathways, and molecular networks, and predicted multiple candidate therapeutic agents. These findings provide a solid theoretical foundation for precision diagnostics and interventions in AIS. However, limitations exist. First, a primary limitation is the relatively small sample size of the metabolomics cohort (10 AIS vs. 10 HC). As an exploratory study, the limited statistical power may affect the sensitivity and generalizability of the identified DEMs. However, to mitigate this limitation, we employed a multi-omics integration strategy, where the metabolomic findings were cross-validated with transcriptomic data from much larger GEO datasets (GSE16561 and GSE58294). The biological consistency observed between the two layers (e.g., in the HIF-1 and FoxO signaling pathways) suggests that the identified metabolites are functionally relevant. Nonetheless, future studies with larger independent cohorts are potentially important to confirm these preliminary findings and evaluate their clinical diagnostic value. Second, although we provided drug predictions and functional annotations for the identified genes, these findings are based on bioinformatics mapping and literature review. Direct functional assays, such as *in vitro* gene knockdown or *in vivo* stroke models, were not performed in the current study to confirm the exact biological roles of these genes or the therapeutic effects of the predicted drugs. Future research will focus on elucidating the specific biological potential pathways of ITGAM and STAT3 in the post-stroke immune response through functional assays to confirm their potential as therapeutic targets. Experimental validation using clinical specimens and animal models is warranted. Future work will incorporate multi-center prospective datasets and focus on functional validation and spatiotemporal dynamics of key genes, to advance personalized therapeutic strategies for stroke patients.

## Data Availability

The datasets presented in this study can be found in online repositories. The names of the repository/repositories and accession number(s) can be found in the article/Supplementary material.

## Footnotes

1
https://www.ncbi.nlm.nih.gov/geo/

2
https://string-db.org/

3
https://CRAN.R-project.org/package=e1071

## References

[1] GBD 2021 Stroke Risk Factor Collaborators. Global, regional, and national burden of stroke and its risk factors, 1990–2021: a systematic analysis for the global burden of disease study 2021. Lancet Neurol. (2024) 23:973–1003. doi: 10.1016/s1474-4422(24)00369-7, 39304265 PMC12254192

[2] Wang X Li A Fan H Li Y Yang N Tang Y. Astrocyte-derived extracellular vesicles for ischemic stroke: therapeutic potential and prospective. Aging Dis. (2024) 15:1227–54. doi: 10.14336/ad.2023.0823-1, 37728588 PMC11081164

[3] Yousufuddin M Young N. Aging and ischemic stroke. Aging (Albany NY). (2019) 11:2542–4. doi: 10.18632/aging.101931, 31043575 PMC6535078

[4] Jacob MA Ekker MS Allach Y Cai M Aarnio K Arauz A . Global differences in risk factors, etiology, and outcome of ischemic stroke in Young adults-a worldwide Meta-analysis: the goal initiative. Neurology. (2022) 98:e573–88. doi: 10.1212/wnl.0000000000013195, 34906974 PMC8829964

[5] Nguyen TN Abdalkader M Fischer U Qiu Z Nagel S Chen HS . Endovascular management of acute stroke. Lancet. (2024) 404:1265–78. doi: 10.1016/s0140-6736(24)01410-7, 39341645

[6] Hilkens NA Casolla B Leung TW de Leeuw FE. Stroke. Lancet. (2024) 403:2820–36. doi: 10.1016/s0140-6736(24)00642-1 38759664

[7] Montaner J Ramiro L Simats A Tiedt S Makris K Jickling GC . Multilevel omics for the discovery of biomarkers and therapeutic targets for stroke. Nat Rev Neurol. (2020) 16:247–64. doi: 10.1038/s41582-020-0350-6, 32322099

[8] Kim Y Cheng W Cho CS Hwang Y Si Y Park A . Seq-scope: repurposing Illumina sequencing flow cells for high-resolution spatial transcriptomics. Nat Protoc. (2025) 20:643–89. doi: 10.1038/s41596-024-01065-0, 39482362 PMC11896753

[9] Tian KJ Yang Y Chen GS Deng NH Tian Z Bai R . Omics research in atherosclerosis. Mol Cell Biochem. (2025) 480:2077–102. doi: 10.1007/s11010-024-05139-1, 39446251

[10] Zhang N Kandalai S Zhou X Hossain F Zheng Q. Applying multi-omics toward tumor microbiome research. iMeta. (2023) 2:e73. doi: 10.1002/imt2.73, 38868335 PMC10989946

[11] Li W Shao C Zhou H Du H Chen H Wan H . Multi-omics research strategies in ischemic stroke: a multidimensional perspective. Ageing Res Rev. (2022) 81:101730. doi: 10.1016/j.arr.2022.101730, 36087702

[12] Ritchie ME Phipson B Wu D Hu Y Law CW Shi W . Limma powers differential expression analyses for Rna-sequencing and microarray studies. Nucleic Acids Res. (2015) 43:e47. doi: 10.1093/nar/gkv007, 25605792 PMC4402510

[13] Wu T Hu E Xu S Chen M Guo P Dai Z . Clusterprofiler 4.0: a universal enrichment tool for interpreting omics data. Innovation (Camb). (2021) 2:100141. doi: 10.1016/j.xinn.2021.100141, 34557778 PMC8454663

[14] Shannon P Markiel A Ozier O Baliga NS Wang JT Ramage D . Cytoscape: a software environment for integrated models of biomolecular interaction networks. Genome Res. (2003) 13:2498–504. doi: 10.1101/gr.1239303, 14597658 PMC403769

[15] Kong C Zhu Y Xie X Wu J Qian M. Six potential biomarkers in septic shock: a deep bioinformatics and prospective observational study. Front Immunol. (2023) 14:1184700. doi: 10.3389/fimmu.2023.1184700, 37359526 PMC10285480

[16] Robin X Turck N Hainard A Tiberti N Lisacek F Sanchez JC . Proc: an open-source package for R and S+ to analyze and compare roc curves. BMC Bioinformatics. (2011) 12:77. doi: 10.1186/1471-2105-12-77, 21414208 PMC3068975

[17] Han D Liu H Gao Y. The role of peripheral monocytes and macrophages in ischemic stroke. Neurol Sci. (2020) 41:3589–607. doi: 10.1007/s10072-020-04777-9, 33009963

[18] Subramanian A Tamayo P Mootha VK Mukherjee S Ebert BL Gillette MA . Gene set enrichment analysis: a knowledge-based approach for interpreting genome-wide expression profiles. Proc Natl Acad Sci USA. (2005) 102:15545–50. doi: 10.1073/pnas.0506580102, 16199517 PMC1239896

[19] Hänzelmann S Castelo R Guinney J. Gsva: gene set variation analysis for microarray and Rna-Seq data. BMC Bioinformatics. (2013) 14:7. doi: 10.1186/1471-2105-14-7, 23323831 PMC3618321

[20] Luo W Brouwer C. Pathview: an R/Bioconductor package for pathway-based data integration and visualization. Bioinformatics. (2013) 29:1830–1. doi: 10.1093/bioinformatics/btt285, 23740750 PMC3702256

[21] Jiang X Zhang R Lu G Zhou Y Li J Jiang X . Brain-derived Exosomal Circrnas in plasma serve as diagnostic biomarkers for acute ischemic stroke. J Neuroimmune Pharmacol. (2024) 19:15. doi: 10.1007/s11481-024-10113-1, 38647743

[22] Yang S Qin C Chen M Chu YH Tang Y Zhou LQ . Trem2-Igf1 mediated Glucometabolic enhancement underlies microglial neuroprotective properties during ischemic stroke. Adv Sci (Weinh). (2024) 11:e2305614. doi: 10.1002/advs.202305614, 38151703 PMC10933614

[23] Sultan R Zou Q Cao Y Hong H Sun R Zhuang C . Mechanisms and implications of mitochondrial autophagy in stroke. Neuropharmacol Ther. (2024) 1:49–64. doi: 10.15212/npt-2024-0005

[24] Fitzgerald KA Kagan JC. Toll-like receptors and the control of immunity. Cell. (2020) 180:1044–66. doi: 10.1016/j.cell.2020.02.041, 32164908 PMC9358771

[25] Qin C Yang S Chu YH Zhang H Pang XW Chen L . Signaling pathways involved in ischemic stroke: molecular mechanisms and therapeutic interventions. Signal Transduct Target Ther. (2022) 7:215. doi: 10.1038/s41392-022-01064-1, 35794095 PMC9259607

[26] Lian L Zhang Y Liu L Yang L Cai Y Zhang J . Neuroinflammation in ischemic stroke: focus on Microrna-mediated polarization of microglia. Front Mol Neurosci. (2020) 13:612439. doi: 10.3389/fnmol.2020.612439, 33488360 PMC7817943

[27] Liu L Xu TC Zhao ZA Zhang NN Li J Chen HS. Toll-like receptor 4 signaling in neurons mediates cerebral ischemia/reperfusion injury. Mol Neurobiol. (2023) 60:864–74. doi: 10.1007/s12035-022-03122-9 36385232

[28] Tang H Zhang Y Liao D Hu J Zhang M Li S . The role of the toll-like receptor family in stroke: functions, mechanisms and therapeutic targets. Brain Res Bull. (2026) 237:111806. doi: 10.1016/j.brainresbull.2026.111806, 41796908

[29] Hou L Li Z Guo X Lv J Chong Z Xiao Y . Itgam is a critical gene in ischemic stroke. Aging. (2024) 16:6852–67. doi: 10.18632/aging.205729, 38637126 PMC11087101

[30] Qiu YM Zhang CL Chen AQ Wang HL Zhou YF Li YN . Immune cells in the Bbb disruption after acute ischemic stroke: targets for immune therapy? Front Immunol. (2021) 12:678744. doi: 10.3389/fimmu.2021.678744, 34248961 PMC8260997

[31] Chen Z Li Q Wang K Yang L Jia Y Gong Z. Brain pericytes—crucial regulators of neuroinflammation in ischemic stroke. Neuropharmacol Ther. (2024) 1:3–17. doi: 10.15212/npt-2024-0004

[32] Wolosowicz M Prokopiuk S Kaminski TW. Matrix metalloproteinase-9 (Mmp-9) as a therapeutic target: insights into molecular pathways and clinical applications. Pharmaceutics. (2025) 17:1425. doi: 10.3390/pharmaceutics17111425, 41304763 PMC12655286

[33] Ha GH Kim EJ Park JS Kim JE Nam H Yeon JY . Jak2/Stat3 pathway mediates neuroprotective and pro-Angiogenic treatment effects of adult human neural stem cells in middle cerebral artery occlusion stroke animal models. Aging (Albany NY). (2022) 14:8944–69. doi: 10.18632/aging.204410, 36446389 PMC9740376

[34] Borbor M Yin D Brockmeier U Wang C Doeckel M Pillath-Eilers M . Neurotoxicity of ischemic astrocytes involves Stat3-mediated metabolic switching and depends on glycogen usage. Glia. (2023) 71:1553–69. doi: 10.1002/glia.24357, 36810803

[35] Adly Sadik N Ahmed Rashed L Ahmed Abd-El Mawla M. Circulating Mir-155 and Jak2/Stat3 axis in acute ischemic stroke patients and its relation to post-ischemic inflammation and associated ischemic stroke risk factors. Int J Gen Med. (2021) 14:1469–84. doi: 10.2147/ijgm.S295939, 33911894 PMC8071708

[36] Chen X Gan Y Zhang K Wu Y Li Y Lan T . Microrna-204-5p deficiency within the Vmpfc region contributes to Neuroinflammation and behavioral disorders via the Jak2/Stat3 signaling pathway in rats. Adv Sci (Weinh). (2025) 12:e2403080. doi: 10.1002/advs.202403080, 39792918 PMC11905084

[37] Zhu H Hu S Li Y Sun Y Xiong X Hu X . Interleukins and ischemic stroke. Front Immunol. (2022) 13:828447. doi: 10.3389/fimmu.2022.828447, 35173738 PMC8841354

[38] Cai W Hu M Li C Wu R Lu D Xie C . Foxp3+ macrophage represses acute ischemic stroke-induced neural inflammation. Autophagy. (2023) 19:1144–63. doi: 10.1080/15548627.2022.2116833, 36170234 PMC10012925

[39] Gao Y Zandieh K Zhao K Khizanishvili N Fazio PD Yu X . The long non-coding Rna Neat1 contributes to aberrant Stat3 signaling in pancreatic cancer and is regulated by a metalloprotease-disintegrin Adam8/Mir-181a-5p axis. Cell Oncol (Dordr). (2024) 48:391–409. doi: 10.1007/s13402-024-01001-0, 39412616 PMC11996950

[40] Wang X Wang Q Wang K Ni Q Li H Su Z . Is immune suppression involved in the ischemic stroke? A study based on computational biology. Front Aging Neurosci. (2022) 14:830494. doi: 10.3389/fnagi.2022.830494, 35250546 PMC8896355

