# Supplementary material for: Identification of key genes and metabolites in acute ischemic stroke via integrated transcriptomic and metabolomic analysis
Source: Front Neurol. 2026 Apr 15;17:1767562. doi: 10.3389/fneur.2026.1767562 (PMC13124469; doi:10.3389/fneur.2026.1767562)
Supplement: Supplementary file 2 [file Table_2.docx]

**Table S2 Demographic and clinical characteristics of acute ischemic stroke (AIS) patients and control subjects**

Supplementary Material

This study enrolled 10 patients with acute ischemic stroke (AIS) and 10 non-stroke controls, with age and sex matched between the two groups. The demographic and clinical baseline characteristics of the subjects are summarized as follows. All **quantitative variables** are presented as **mean ± standard error of the mean (SEM)**, and **categorical variables** as **number (percentage)**. P values were calculated using Fisher's exact test (for categorical variables) or Student's t-test (for quantitative variables), with the test criteria referenced to the clinical diagnostic specifications for risk factors of ischemic stroke.

## Notes

1. Abbreviations: BMI = Body Mass Index; HDL = High-density lipoprotein; LDL = Low-density lipoprotein; Lp(a) = Lipoprotein(a); NIHSS = National Institutes of Health Stroke Scale; AIS = Acute ischemic stroke.
2. Diagnostic criteria: ① Hypertension: Blood pressure ≥140/90 mmHg; ② Diabetes mellitus: Fasting blood glucose ≥7.0 mM or 2-hour postprandial blood glucose ≥11.1 mM.
3. Unit conversion: The original data of lipoprotein(a) were partially in mg/L and uniformly converted to g/L (conversion factor: 1 g/L = 1000 mg/L) to ensure the consistency of data units.
4. Sample size description: A total of 10 cases were included in both the control group and the AIS group. The standard error of the mean (SEM) for quantitative variables was calculated according to the formula: SEM = SD/√n (n=10, √10≈3.1623).
5. NIHSS score: The National Institutes of Health Stroke Scale score was only assessed in the AIS group, with no available results for the control group.
6. TOAST classification: The TOAST classification was only assessed in the AIS group, with no available results for the control group.

| **Characteristics** | **Control (n=10)** | **AIS (n=10)** | **P** |
| --- | --- | --- | --- |
| Age (years) | 65.90 ± 1.82 | 65.70 ± 2.05 | 0.905 |
| Sex (male), n (%) | 5(50.0) | 7(70.0) | 0.647 |
| BMI (kg/m²) | 23.89 ± 0.52 | 22.08 ± 1.12 | 0.215 |
| Smoking, n (%) | 3(30.0) | 5(50.0) | 0.408 |
| Alcohol consumption, n (%) | 0(0.0) | 3(30.0) | 0.214 |
| Hypertension, n (%) | 1(10.0) | 7(70.0) | 0.012 |
| Diabetes mellitus, n (%) | 2(20.0) | 4(40.0) | 0.609 |
| Total cholesterol (mmol/L) | 3.60 ± 0.32 | 3.64 ± 0.25 | 0.930 |
| Triglycerides (mmol/L) | 1.13 ± 0.14 | 1.38 ± 0.22 | 0.352 |
| LDL (mmol/L) | 2.40 ± 0.20 | 2.19 ± 0.17 | 0.457 |
| HDL (mmol/L) | 1.59 ± 0.17 | 1.20 ± 0.13 | 0.028 |
| Lp(a) (g/L) | 0.093 ± 0.019 | 0.126 ± 0.025 | 0.266 |
| NIHSS score | — | — | — |
| 4–9 | — | 8(80.0%) | — |
| 10–14 | — | 1(10.0%) | — |
| 15–19 | — | 1(10.0%) | — |
| 20–25 | — | 0(0.0%) | — |
| TOAST classification, n (%) |  |  |  |
| Large artery atherosclerosis |  | 4(40.0) | - |
| Cardioembolism |  | 3(30.0) | - |
| Small artery occlusion |  | 3(30.0) | - |
